# Comparative Analysis of Laparoscopic Versus Open Surgery in Colorectal Cancer: An Eight-Year Single-Center Experience From Jordan

**DOI:** 10.7759/cureus.73746

**Published:** 2024-11-15

**Authors:** Haitham S Rbeihat, Abdullah A Abu Anzeh, Ruba Y Shannaq, Mohammad A Abu Alanaz, Alaa M Khamaiseh, Ghaseb A Abu Alghawai, Mahmoud Swalqa, Muhannad F Lababneh, Ali ALoun, Majed Alqaisi, Ahmad A Uraiqat

**Affiliations:** 1 Department of General Surgery, King Hussein Medical Center, Royal Medical Services, Amman, JOR

**Keywords:** colorectal cancer, complications, jordan, laparoscopic surgery, open surgery

## Abstract

Introduction

The use of laparoscopic surgery has increased in the treatment of colorectal cancer (CRC). However, achieving oncological outcomes similar to those of open surgery remains challenging, particularly for CRC. In this comparative, retrospective study, we aim to investigate and compare the postoperative complications of open and laparoscopic CRC surgery in Jordan.

Methods

Using a retrospective study design, patients’ records were obtained from the electronic hospital database of King Hussein Medical Center, Amman, Jordan, during the period between 2016 and 2024. Demographic data were collected for age and gender. Clinical data were collected for tumor site, tumor grade, body mass index, American Society of Anesthesiologists (ASA) score, lymph node ratio (LNR), postoperative complications such as ileus, anastomosis, stoma, renal complications, pain, wound infection, and death, and length of hospital stay.

Results

We included 857 CRC patients, with 437 (51.0%) undergoing laparoscopic resection and 420 (49%) undergoing open resection. The mean age was 58 years, with no age difference between the study groups. Most patients (507, 59%) were in good health based on the ASA score. The majority (671, 78%) had moderately differentiated tumors, with 320 (76%) in the open surgery group and 351 (80%) in the laparoscopy group. The mean LNR was 0.19, trending higher in the group that underwent open surgery (0.33 vs. 0.09, p = 0.065). The open surgery group had a significantly longer hospital stay (5.28 days) relative to the laparoscopic group (3.77 days, p < 0.001). Postoperative complications included wound infection (33, 3.9%), ileus (19, 2.2%), stoma (15, 1.8%), anastomosis (10, 1.2%), renal complications (9, 1.1%), and pain (6, 0.7%). The mortality rate was higher in the open surgery group (p = 0.035). Most patients (711, 83%) did not experience postoperative complications.

Conclusion

This is the first Jordanian study to compare long-term outcomes of CRC patients undergoing open versus laparoscopic surgical resection. Our findings suggested that the laparoscopic group had a shorter hospital stay, with no differences in postoperative complications rate between the study groups. Mortality rates were low overall but significantly higher in the open surgery group. These results suggest that laparoscopic resection may be superior for CRC surgery, though further multicenter studies are warranted to confirm our findings.

## Introduction

Colorectal cancer (CRC) is the second most common cause of death related to cancer globally after lung cancer, accounting for 935,173 total deaths in 2020. Regarding its incidence, CRC is the third most common cancer, and the global incidence rate is 19.5:100,000 person-year [1,2]. The latest update of the American Cancer Society on CRC statistics showed that the overall yearly incidence of CRC has decreased from 1985 to 2019 by 46%, which was due to lifestyle modifications and CRC screening programs [3]. According to the Jordan Cancer Registry, in 2018, CRC ranked as the second most prevalent cancer type among both males and females, representing 10.6% of all cancer cases [4].

CRC has several risk factors, including modifiable and non-modifiable factors. Modifiable CRC risk factors include smoking, chronic alcohol consumption, unhealthy lifestyle, obesity, and stress [5]. Whereas advanced age, male gender, and genetic conditions, such as familial adenomatous polyposis and Lynch syndrome, are among the non-modifiable CRC risk factors [6,7]. Primary CRC prevention includes risk factor elimination and lifestyle modifications [8,9]. Secondary prevention includes early CRC screening, which aims at diagnosing and treating premalignant CRC lesions such as colorectal polyps [7,10].

Surgical resection is the first-line treatment in patients with non-metastatic CRC proceeded by adjuvant chemotherapy. After surgical resection of tumor margins and regional lymph nodes, the resected lesions are assessed for pathological staging [11]. The first laparoscopic colectomy was introduced in 1991, raising a lot of controversies regarding the technical difficulties of this technique, and its oncological outcomes [12]. With the advancement of technology, it has become more widely used, and several randomized controlled trials (RCTs) showed comparable oncological outcomes between open and laparoscopic approaches of colectomy [13,14]. Complications after CRC surgery vary between intraoperative complications, such as bleeding and organ injury, and postoperative complications, such as infections, anastomosis, and ileus [15].

Many studies have shown the superiority of laparoscopic colectomy in short-term postoperative outcomes [16,17]. However, there is still a controversy regarding the long-term outcomes of laparoscopic colectomy compared to open surgery. Therefore, in this retrospective, comparative study, we aim to investigate the long-term outcomes and complication rates between laparoscopic versus open colectomy for resectable CRC patients in the Jordanian population over an eight-year period.

## Materials and methods

Study design

We carried out a single-center, retrospective comparative study at King Hussein Medical Center (KHMC) in Jordan between the period of May 2016 to April 2024. Our inclusion criteria consisted of patients diagnosed with CRC and who had undergone surgery and adjuvant chemotherapy.

Patients were first grouped into either colonic or rectal cancer, and computed tomography (CT) scans for the abdomen and chest were obtained, along with colonoscopy and biopsy to determine the stage of the tumor. After staging and tumor marker analysis (carcinoembryonic antigen, CEA), a team of oncology experts discussed the respectability of the tumor to determine the treatment approach. Patients with unresectable tumors underwent palliative chemotherapy and were excluded from this study. Patients with resectable rectal cancer underwent neoadjuvant chemo- and radiotherapy, and patients with advanced-stage resectable colon cancer underwent neoadjuvant chemotherapy. Early-stage resectable colon cancer patients underwent surgical resection. Patients were then followed on the Enhanced Recovery After Surgery (ERAS) protocol.

Study outcomes and data collection

The primary outcome of our study was the mortality rate defined as the number of deaths after surgery. Secondary outcomes included hospitalization period and postoperative complications like ileus, anastomosis, wound infection, stoma, pain, and renal complications like kidney failure. By including these specific complications, we aimed to provide clinically relevant insights to guide surgical decision-making in CRC management. Data were retrospectively collected by accessing the hospital’s medical records from May 2016 to April 2024. The following demographic and clinical variables were collected: patient’s hospital identity (ID), national ID, age, sex, American Society of Anesthesiologists (ASA) score, tumor grade, the number of positive lymph nodes (LNs), the total number of harvested LNs, and the lymph node ratio (LNR) calculated as the ratio between the number of positive LNs and the total number of LNs. After removing records with missing information, a total of 857 CRC patients who underwent either open or laparoscopic surgical resection were included.

Ethical approval

The Institutional Review Board at King Hussein Medical Center granted ethical approval for this study. Since this was a retrospective study using de-identified data from existing medical records, the need for informed consent was waived in agreement with the Declaration of Helsinki. All procedures adhered to relevant ethical standards and institutional guidelines.

Statistical analysis

Continuous data were summarized using means (standard deviation) and analyzed with the Student’s t-test for normally distributed data, and medians (range) with the Wilcoxon rank-sum test for non-normally distributed data. Categorical variables were described using frequencies and percentages and analyzed with the chi-square and Fisher's exact test. For comparisons between more than two groups, the one-way analysis of variance (ANOVA) test was utilized, and for non-normally distributed data, the Kruskal-Wallis test was employed. A logistic regression model was constructed to examine the association between the type of surgery and postoperative complications. A p-value of <0.05 was considered to be statistically significant. All analyses were performed using R statistical software (R Foundation for Statistical Computing, Vienna, Austria).

## Results

We included a total of 857 CRC patients from KHMC, of whom 437 (51.0%) underwent laparoscopic surgical resection, and 420 (49%) underwent open resection. The mean age was 58.0 (14.0) years, with no significant difference between the laparoscopy and open surgery groups (p-value > 0.05). Males accounted for 57% of the study samples, and 232 (55%) males had open surgery while 253 (58%) had laparoscopy (Table 1). The majority of patients (59%) were in good health based on the ASA score, and 34% had mild systemic diseases (ASA score = 2). In patients who underwent open surgery, 269 (64%) were in good health (ASA score = 1), while 164 (38%) in the laparoscopy group had mild systemic diseases, and 33 (7.6%) had severe systemic diseases (ASA score = 3) (p-value = 0.021). The majority of samples (78%) had moderately differentiated tumors, and 71 (8.3%) had invasive moderately differentiated adenocarcinoma (IMDA). In the open surgery group, 320 (76%) had moderately differentiated tumors, compared to 351 (80%) in the laparoscopy group. The mean LNR was 0.19 (2.05), with a trend toward a higher ratio in the open surgery group (mean LNR: 0.33 (3.06) vs. 0.09 (0.16), p-value = 0.065).

**Table 1:** Comparison between multiple surgical approaches. ^*^ n (%); mean (SD); ^# ^Pearson’s chi-squared test; ^% ^Kruskal-Wallis rank sum test; ^^ ^Fisher’s exact test. Lap into open: patients who were originally appointed for laparoscopy and converted to open surgery mid-procedure; ASA: American Society of Anesthesiologists; LN: lymph node; LNR: lymph node ratio (positive LNs/total LNs dissected); IMDA: invasive moderately differentiated adenocarcinoma.

Characteristic	Laparoscopy (N^*^ = 437)	Open (N^*^ = 368)	Lap into open (N^*^ = 52)	p-value	Overall (N^*^ = 857)
Gender				0.5^#^	
Male	253 (58%)	200 (54%)	32 (62%)		485 (57%)
Female	184 (42%)	168 (46%)	20 (38%)		372 (43%)
Age	58 ± 14	58 ± 13	60 ± 13	0.5^%^	58 ±14
ASA score				NA	
1	238 (54%)	238 (65%)	31 (60%)		507 (59%)
2	164 (38%)	110 (30%)	17 (33%)		291 (34%)
3	33 (7.6%)	20 (5.4%)	4 (7.7%)		57 (6.7%)
4	1 (0.2%)	0 (0%)	0 (0%)		1 (0.1%)
5	1 (0.2%)	0 (0%)	0 (0%)		1 (0.1%)
Tumor grade				NA	
Moderately differentiated	351 (80%)	283 (77%)	37 (71%)		671 (78%)
IMDA	29 (6.6%)	38 (10%)	4 (7.7%)		71 (8.3%)
Well-differentiated	38 (8.7%)	21 (5.7%)	9 (17%)		68 (7.9%)
Poorly differentiated	19 (4.3%)	26 (7.1%)	2 (3.8%)		47 (5.5%)
Positive LNs	2.2 ± 4.0	3.2 ± 6.7	4.8 ± 9.1	0.6^%^	2.8 ±5.7
Total LNs	26 ± 16	23 ± 19	25 ± 11	0.019^%^	25 ±17
LNR	0.09 ± 0.16	0.35 ± 3.30	0.19 ± 0.30	0.2^%^	0.19 ±2.05
Hospital stays (days)	3.77 ± 3.27	5.41 ± 3.93	4.50 ± 2.41	<0.001^%^	4.47 ±3.59
Postoperative complications					
Ileus	8 (1.8%)	9 (2.4%)	2 (3.8%)	0.4^^^	19 (2.2%)
Anastomosis	5 (1.1%)	4 (1.1%)	1 (1.9%)	0.7^^^	10 (1.2%)
Stoma	7 (1.6%)	5 (1.4%)	3 (5.8%)	0.11^^^	15 (1.8%)
Renal	6 (1.4%)	3 (0.8%)	0 (0%)	0.7^^^	9 (1.1%)
Death	1 (0.2%)	6 (1.6%)	1 (1.9%)	0.058^^^	8 (0.9%)
Pain	2 (0.5%)	2 (0.5%)	2 (3.8%)	0.07^^^	6 (0.7%)
Wound infection	17 (3.9%)	12 (3.3%)	4 (7.7%)	0.3^^^	33 (3.9%)
None	361 (83%)	315 (86%)	35 (67%)	0.004^#^	711 (83%)

The mean length of hospital stay was 4.47 (3.59) days, with a significantly longer duration of stay in the open surgery group (mean: 5.28 (3.77) vs. 3.77 (3.27), p-value < 0.001). Postoperative complications included wound infection in 33 (3.9%), ileus in 19 (2.2%), stoma in 15 (1.8%), anastomosis in 10 (1.2%), renal complications in nine (1.1%), pain in six (0.7%), and eight (0.9%) died, of whom seven were from the open surgery group compared to one in the laparoscopy group (p-value = 0.035). The majority of patients (83%) did not experience postoperative complications, as shown in Table 2.

**Table 2:** Comparison between laparoscopy converted to open and open surgical approaches. ^* ^n (%); mean ± SD; ^# ^Pearson’s chi-squared test; ^% ^Wilcoxon rank-sum test; ​​​​​​​^^ ^Fisher’s exact test. ASA: American Society of Anesthesiologists; LN: lymph node; LNR: lymph node ratio (positive LNs/total LNs dissected); IMDA: invasive moderately differentiated adenocarcinoma.

Characteristic	Laparoscopy (N^*^ = 437)	Open (N^*^ = 420)	p-value	Overall (N^*^ = 857)
Gender			0.4^#^	
Male	253 (58%)	232 (55%)		485 (57%)
Female	184 (42%)	188 (45%)		372 (43%)
Age	58 ± 14	58 ± 13	>0.9^%^	58 ±14
ASA score			0.021^^^	
1	238 (54%)	269 (64%)		507 (59%)
2	164 (38%)	127 (30%)		291 (34%)
3	33 (7.6%)	24 (5.7%)		57 (6.7%)
4	1 (0.2%)	0 (0%)		1 (0.1%)
5	1 (0.2%)	0 (0%)		1 (0.1%)
Tumor grade			0.1^#^	
Moderately differentiated	351 (80%)	320 (76%)		671 (78%)
IMDA	29 (6.6%)	42 (10%)		71 (8.3%)
Well-differentiated	38 (8.7%)	30 (7.1%)		68 (7.9%)
Poorly differentiated	19 (4.3%)	28 (6.7%)		47 (5.5%)
Positive LNs	2.2 ± 4.0	3.4 ± 7.1	0.4^%^	2.8 ± 5.7
Total LNs	26 ± 16	23 ± 18	0.018^%^	25 ± 17
LNR	0.09 ± 0.16	0.33 ± 3.06	0.065^%^	0.19 ± 2.05
Hospital stays (days)	3.77 ± 3.27	5.28 ± 3.77	<0.001^%^	4.47 ± 3.59
Postoperative complications				
Ileus	8 (1.8%)	11 (2.6%)	0.4^#^	19 (2.2%)
Anastomosis	5 (1.1%)	5 (1.2%)	>0.9^#^	10 (1.2%)
Stoma	7 (1.6%)	8 (1.9%)	0.7^#^	15 (1.8%)
Renal	6 (1.4%)	3 (0.7%)	0.5^^^	9 (1.1%)
Dead	1 (0.2%)	7 (1.7%)	0.035^^^	8 (0.9%)
Pain	2 (0.5%)	4 (1.0%)	0.4^^^	6 (0.7%)
Wound infection	17 (3.9%)	16 (3.8%)	>0.9^#^	33 (3.9%)
None	361 (83%)	350 (83%)	0.8^#^	711 (83%)

Logistic regression analysis for postoperative complications showed a significant association with sex, in which males had a 1.8 times higher likelihood of postoperative complications compared to females (OR: 1.81, 95% CI: 1.09 to 3.02, p-value = 0.023). No significant association between postoperative complications and age was found (p-value > 0.05). Tumor grade was significantly associated with postoperative complications, in which patients with moderately differentiated tumors had a higher likelihood of having postoperative complications (OR: 4.33, 95% CI: 1.28 to 14.68, p-value = 0.019). In addition, longer duration of hospital stay was associated with a higher likelihood of postoperative complications (OR: 1.29, 95% CI: 1.18-1.40, p-value < 0.001). The number of positive LNs was associated with higher rates of postoperative complications (OR: 1.09, 95% CI: 1.01 to 1.18, p-value = 0.034) with no significant association with LNR and mode of surgery, as shown in Table 3.

**Table 3:** Logistic regression model for the association between mode of surgery and study variables. LN: lymph node; LNR: lymph node ratio (positive LNs/total LNs dissected); IMDA: invasive moderately differentiated adenocarcinoma.

Predictor	Odds ratio		95% CI		p-value
			Lower	Upper	
Gender					
Male – female		1.8095	1.08522	3.0171	0.023
Age		0.9996	0.98163	1.018	0.969
Tumor grade					
Moderately differentiated – IMDA		4.327	1.27582	14.6752	0.019
Poorly differentiated – IMDA		1.373	0.24809	7.5986	0.716
Well-differentiated – IMDA		3.369	0.81678	13.8958	0.093
Hospital stays (days)		1.2851	1.18162	1.3976	
LNR		0.1934	0.01817	2.0584	0.173
Positive LNs		1.0881	1.00658	1.1763	0.034
Mode of surgery					
Open – laparoscopy		0.6919	0.40829	1.1725	0.171

## Discussion

According to the GLOBOCAN 2012 study, Jordan was the second highest country in the Middle Eastern region in CRC incidence rate and had the highest mortality rate [18,19]. Screening and early prevention efforts have reduced CRC incidence and mortality rates, yet few people undergo early screening tests, and most patients are diagnosed at the late stages of the disease [20]. The standard management protocol for patients diagnosed with CRC is surgical resection. However, there is no consensus on the choice between laparoscopy versus open surgical resection. Therefore, in this comparative, retrospective study, we aim to compare the postoperative outcomes from complications and hospital stays among CRC patients who underwent laparoscopy or open surgical resection in the Jordanian population.

Our study included a total of 857 CRC patients, grouped into laparoscopic resection (n = 437) and open resection (n = 420). There was no sex predominance in our cohort. The mean age was 58 years in both study groups. Recently, there was a significant decline in CRC incidence in older people due to screening efforts; however, a concurrent increase in CRC incidence was seen in younger people [21]. In developing nations such as Jordan, CRC screening rates are significantly lower than in developed nations due to several barriers, including patient-related barriers such as patients’ awareness and having no complaints, in addition to healthcare-related barriers, which included access to healthcare screening facilities and physician endorsements [22,23]. In Jordan, a cross-sectional study by Jadallah et al. showed that 41.7% of participants were aware of CRC screening necessity, but only 17% of participants had CRC screening [19].

Surgical resection is the primary treatment for patients with non-metastatic CRC, typically stages 0-II, with adjuvant chemotherapy recommended for stage II patients. Treatment options for advanced stages and metastatic CRC patients include surgery and neoadjuvant chemotherapy and clinical trials for immunotherapy [24]. Recently, laparoscopic surgery rates increased in comparison to open surgery for both cancerous and non-cancerous colorectal surgery [25]. However, there is still an ongoing debate about whether the outcome of laparoscopic surgery adheres to oncologic principles compared to those of open surgery [26]. In our study, we compared the postoperative complications between laparoscopic and open surgical resection. Our findings showed a significantly longer duration of hospital stay in the open surgery group, compared to those who underwent laparoscopy and who underwent laparoscopy and were converted to open surgery. Similarly, the findings from Chiu et al.'s study also showed a significantly longer hospitalization period in open resection compared to laparoscopy [26]. The pooled analysis of a systematic review and meta-analysis showed a significantly shorter hospital stay in the laparoscopy group compared to open surgery in four studies [27].

Regarding postoperative complications, the majority of patients did not experience complications. However, eight people died, seven of whom had open surgery (p-value < 0.05). When comparing patients who underwent laparoscopy to those who underwent open surgery, there was no significant association with postoperative complications. Interestingly, a longer hospitalization period was associated with postoperative complications, and moderately differentiated tumors were associated with higher postoperative complications compared to invasive moderately differentiated tumors. In a systematic review and meta-analysis by Warps et al., the results showed significantly lower mortality rates and postoperative complications, including wound infection, ileus, and length of hospital stay. However, these outcomes were reported for the short-term, and no significant differences in the long-term outcomes, including overall survival and recurrence-free survival, were seen [16]. Additionally, a network meta-analysis comparing laparoscopy, robot-assisted, and open surgical resection showed no significant difference between the three approaches in blood loss and postoperative complications [28]. A randomized trial by Bonjer et al. compared laparoscopic versus open rectal cancer surgery and showed no significant difference between the two groups in disease-free and overall survival rates (74.8% and 86.7% in the laparoscopy group vs. 70.8% and 83.6% in the open surgery group) [29]. Laparoscopy can show superiority to open surgery in visualizing narrow spaces such as the lower pelvis and gives a better view of the operative field, which is crucial for adequate resection of tumor margins [30].

Our findings should be interpreted with caution due to several limitations. First, we included patients from a single center in Jordan, which may affect the generalizability of our findings. Further multi-center longitudinal studies are needed to evaluate the results across different regions. Second, we implemented a retrospective study design, which can infer the temporal relationship between events. Prospective studies with long-term follow-up periods are needed to evaluate the long-term outcomes between the surgical groups. Despite limitations, this study has notable strengths. To date, this is the first Jordanian study to evaluate the long-term outcomes over a period of 10 years between laparoscopy and open CRC surgery. In addition, our cohort included a substantially large sample size, which increases the power of our results.

## Conclusions

In conclusion, this retrospective study is the first in Jordan to compare the long-term outcomes of CRC patients who underwent laparoscopic versus open surgical resection. Our results demonstrated a significantly shorter hospitalization period for the laparoscopy group, without significant differences in postoperative complications between the two surgical approaches. However, the open surgery group showed higher morbidity rates, including postoperative mortality. These findings indicate that laparoscopic resection may offer advantages over open surgery, particularly in reducing hospital stay and morbidity. Further multicenter studies are necessary to confirm these results and provide broader generalizability across different populations.
